# Atypical *Mycobacterium ulcerans* Infection with Skip Lesions in a 68-Year-Old Male: A Rare Case and Comprehensive Literature Review

**DOI:** 10.3390/jcm14113853

**Published:** 2025-05-30

**Authors:** Roberto Cuomo, Ishith Seth, Gianluca Marcaccini, Phil Y. J. Lu, Warren M. Rozen, Daniel P. O’Brien

**Affiliations:** 1 Department of Plastic and Reconstructive Surgery, University of Siena, 53100 Siena, Italy; gianlu32@gmail.com; 2 Faculty of Medicine and Surgery, Peninsula Clinical School, Monash University, Clayton, VIC 3199, Australia; ishithseth1@gmail.com (I.S.); phillu97@gmail.com (P.Y.J.L.); warrenrozen@hotmail.com (W.M.R.); 3 Department of Plastic and Reconstructive Surgery, Peninsula Health, Frankston, VIC 3199, Australia; 4 Department of Infectious Diseases, Barwon Health, Geelong, VIC 3022, Australia

**Keywords:** *Mycobacterium ulcerans*, Buruli ulcer, skip lesions, necrotizing skin infection, polymerase chain reaction, rifampicin, clarithromycin, surgical debridement, atypical presentation, Australia

## Abstract

**Background/Objectives:** *Mycobacterium ulcerans* causes Buruli ulcers, typically manifesting as a single progressive necrotizing skin lesion. Rarely, non-contiguous “skip lesions” occur, complicating diagnosis and management. This report describes an atypical case and reviews similar presentations to emphasize early recognition and combined therapeutic strategies. **Methods:** A systematic literature review was performed using PubMed, Embase, Cochrane Library, Google Scholar, and Scopus databases until December 2024, focusing on cases involving skip lesions. Additionally, a detailed clinical case of a 68-year-old male from Mornington Peninsula, Australia, presenting skip lesions from the distal leg to the proximal knee was documented, including diagnostic PCR testing, surgical interventions, and antibiotic treatments. **Results:** Twelve studies were identified, totaling 1828 cases with 1179 exhibiting skip lesions. The majority achieved successful outcomes through combined antibiotic (rifampicin and clarithromycin) and surgical management. The reported case initially underwent surgical excision without antibiotics, leading to recurrence. Subsequent comprehensive management combining additional surgical debridement and adjusted antibiotics successfully resolved the infection. **Conclusions:** Although rare, skip lesions significantly complicate the clinical management of Buruli ulcers. Early diagnosis and a multidisciplinary approach integrating surgical debridement and antibiotic therapy are critical for optimal outcomes and minimizing recurrence risks.

## 1. Introduction

*Mycobacterium ulcerans* (*M. ulcerans*) is a slow-growing environmental mycobacterium responsible for Buruli ulcers, also known as Bairnsdale, Daintree, Mossman, Kumasi, or Searls ulcers, which manifest as progressive necrotizing skin and soft-tissue ulcerations [1]. This pathogen is unique in its production of mycolactone, a polyketide-derived macrolide toxin pivotal in disease pathogenesis, causing cellular apoptosis and immune system inhibition [2]. The ability of mycolactone to suppress local immune responses allows the infection to persist and spread subcutaneously, leading to the characteristic painless ulcers with extensive necrosis. If left untreated, *M. ulcerans* infections can result in deformities, functional disabilities, and, in rare cases, systemic complications [1]. Unlike other mycobacterial infections, such as those caused by *Mycobacterium tuberculosis* or *Mycobacterium leprae*, *M. ulcerans* infections typically remain localized to the skin and subcutaneous tissue. However, in some instances, such as the one presented here, skip lesions suggest a more complex dissemination pattern [1].

Buruli ulcer is considered a neglected tropical disease and is endemic in tropical and subtropical regions, particularly West and Central Africa, where it represents a significant public health burden [3]. The disease is also well documented in parts of South America and Asia. However, in recent years, there has been a notable rise in cases in temperate regions, particularly in southeastern Australia. Victoria has emerged as an important endemic area, with increasing numbers of cases reported along the coastal regions of the Mornington and Bellarine Peninsulas [3]. Urban centers such as Geelong and Melbourne have documented cases, indicating a progressive expansion of the disease’s geographic range [3].

The exact transmission mode of *M. ulcerans* remains unclear, but it is believed to occur through environmental exposure rather than direct human-to-human transmission. Multiple ecological factors, including wetlands, stagnant water, mosquitoes, and possum feces, have been implicated as potential reservoirs and vectors of infection [4,5]. In Australia, *M. ulcerans* DNA has been identified in possum feces, and research suggests that mosquitoes may act as mechanical vectors for transmission to humans. Despite these findings, the full transmission cycle is yet to be fully elucidated [3,6].

The clinical spectrum of *M. ulcerans* infections varies widely depending on disease progression, host immune response, and time to diagnosis. Early-stage infections often present as small, painless nodules or plaques, which may remain undetected for weeks or months [6]. As the disease progresses, the nodules ulcerate, leading to extensive tissue necrosis and ulcer formation. More severe infections can result in edematous lesions that are large, rapidly progressive, and associated with pain, extensive tissue destruction, and functional impairment [7]. Due to its slow progression and relatively painless nature, Buruli ulcer is often misdiagnosed as vascular causative ulcer or other dermatologic conditions, particularly in non-endemic areas, leading to delays in treatment [7].

The standard treatment for *M. ulcerans* involves a combination of antibiotics, typically rifampicin and clarithromycin, administered for eight weeks [8]. Surgical excision is considered in cases where antibiotics alone are insufficient, or rapid tissue debridement is necessary. Wide surgical excision of small *M. ulcerans* lesions has been reported to achieve a 95% cure rate, provided that histological margins are free of inflammation and necrosis [9].

While most patients with *M. ulcerans* present with a single ulcerative lesion, approximately 5% of cases exhibit multiple non-contiguous lesions, known as skip lesions [10]. The mechanism underlying the development of skip lesions is poorly understood, though several theories have been proposed, including lymphatic spread, hematogenous dissemination, or multiple sites of primary inoculation. Some researchers hypothesize that skip lesions may result from a paradoxical immune response, where subclinical lesions become apparent after the immune system mounts a reaction against the primary site of infection. Given the progressive increase in cases of Buruli ulcer in temperate regions, recognizing skip lesions as a distinct and potentially underreported manifestation of *M. ulcerans* is critical [8,9,10,11].

This case report describes an unusual presentation of Buruli ulcer in a 68-year-old male from the Mornington Peninsula, Australia, who developed skip lesions extending from the distal leg to the proximal knee. The atypical non-contiguous lesions posed a diagnostic challenge despite being in an endemic region. This report highlights the importance of the early recognition and appropriate management of skip lesions. It includes a systematic literature review of similar cases to explore possible pathophysiologic mechanisms and treatment strategies.

## 2. Materials and Methods

A systematic literature review was conducted to identify and analyze relevant data on *Mycobacterium ulcerans* infections, focusing on cases involving skip lesions and the prevalence of the disease in endemic regions such as the Mornington Peninsula, Australia. The databases searched included PubMed (Medline), Embase, Cochrane Library, Google Scholar, and Scopus, including all works up to December 2024. A structured combination of Medical Subject Heading (MeSH) terms and relevant keywords was used to ensure a comprehensive search. The primary search terms included *Mycobacterium ulcerans*, Buruli ulcer, Bairnsdale ulcer, skip lesions, necrotizing skin infections, Victoria Australia, Mornington Peninsula, prevalence, treatment, rifampicin and clarithromycin, and surgical debridement. Boolean operators such as AND and OR were applied to refine the search strategy and capture all relevant studies.

The inclusion criteria for the literature search encompassed studies that reported on *M. ulcerans* infections presenting with skip lesions, epidemiological studies examining the prevalence of *M. ulcerans* in endemic regions, including Australia, and clinical studies detailing treatment approaches, particularly antibiotic therapy with rifampicin and clarithromycin and the role of surgical debridement. Only peer-reviewed articles published in English were analyzed. Studies that did not meet these criteria, as well as non-English publications, non-peer-reviewed articles, and opinion pieces, were excluded from the review. The screening process involved an initial review of article titles and abstracts, followed by a full-text evaluation of potentially relevant studies. References from selected articles were also assessed to identify further studies that met the inclusion criteria.

To ensure ethical standards, written informed consent was obtained from the patient to publish this case report, including the use of clinical data and accompanying images. The patient was provided with a detailed explanation of the case report’s purpose, the scope of publication, and the measures taken to maintain confidentiality. All identifying information has been removed to comply with privacy regulations. This study was conducted in accordance with the ethical principles outlined in the Declaration of Helsinki and relevant institutional guidelines governing case report publications.

### Case Presentation

**Figure 1:**
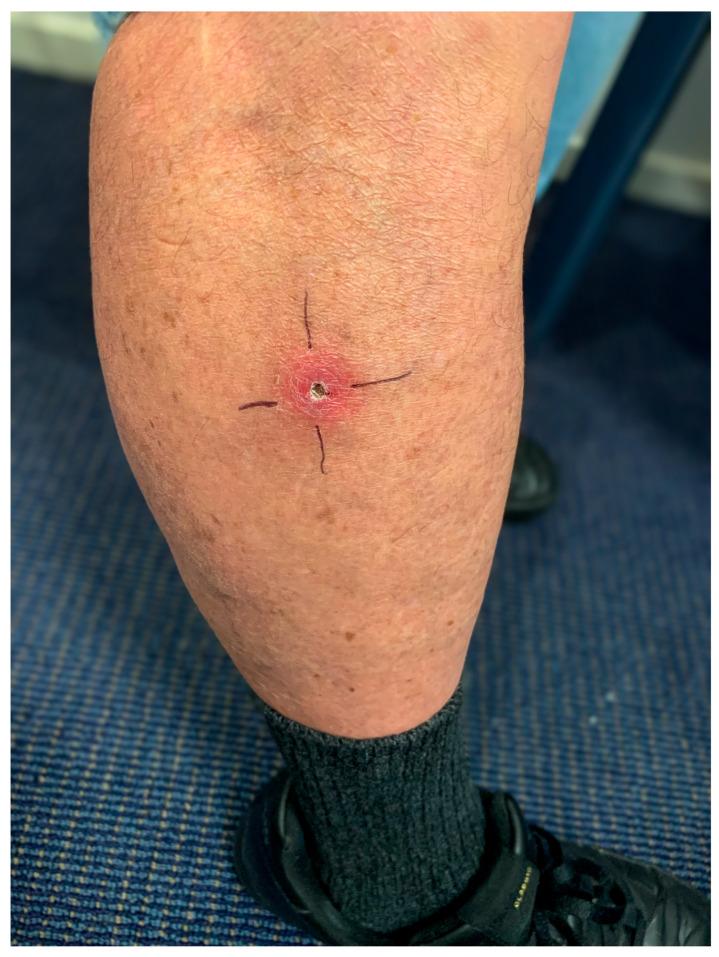
Clinical photograph of initial *M. ulcerans* ulcer on right lateral calf.

A 68-year-old male from the Mornington Peninsula, Victoria, was referred to a Plastic and Reconstructive Surgeon with a recurrent ulcerative lesion on his left knee, following a previously treated *Mycobacterium ulcerans* infection seven months earlier. The initial lesion had been located on the right lateral mid-calf (Figure 1). It was managed with wide local excision without antibiotic therapy due to multiple medical comorbidities and concerns about potential drug interactions. The wound had healed completely for four months postoperatively, with no signs of residual infection.

The World Health Organization (WHO) classifies Buruli ulcer lesions into three categories based on size, number, and anatomical location. Category I lesions are defined as single small lesions less than 5 cm in diameter, Category II includes non-ulcerative or ulcerative lesions between 5 and 15 cm, and Category III comprises lesions greater than 15 cm, multiple lesions, lesions at critical sites (e.g., face, genitalia), or those associated with osteomyelitis. In October 2022, the patient presented with a Category I Buruli ulcer, consisting of a solitary ulcerative lesion approximately 3 cm in diameter affecting the right anterolateral mid-calf.

The patient had a history of atrial fibrillation, for which he was on flecainide (50 mg twice daily), apixaban (5 mg daily), and metoprolol (25 mg twice daily), and had a permanent pacemaker. His past medical history also included hypertension, hypercholesterolemia, stage 3 chronic kidney disease, and gastroesophageal reflux disease, all well controlled with medication. He had previously undergone a total prostatectomy for prostate malignancy. Despite these comorbidities, he remained fit, active, and fully independent in all activities of daily living.

His initial presentation in October 2022 involved a category one Buruli ulcer affecting the right anterolateral mid-calf. A wound swab sent for *M. ulcerans* PCR returned positive. Given his complex medical background and the risk of potentially fatal cardiac arrhythmias associated with rifampicin and clarithromycin interactions with his usual medication, the decision was made to proceed with wide surgical excision alone, without antibiotic therapy. Histological examination confirmed clear margins free of necrotic tissue or inflammatory involvement. The wound healed uneventfully over four months.

**Figure 2:**
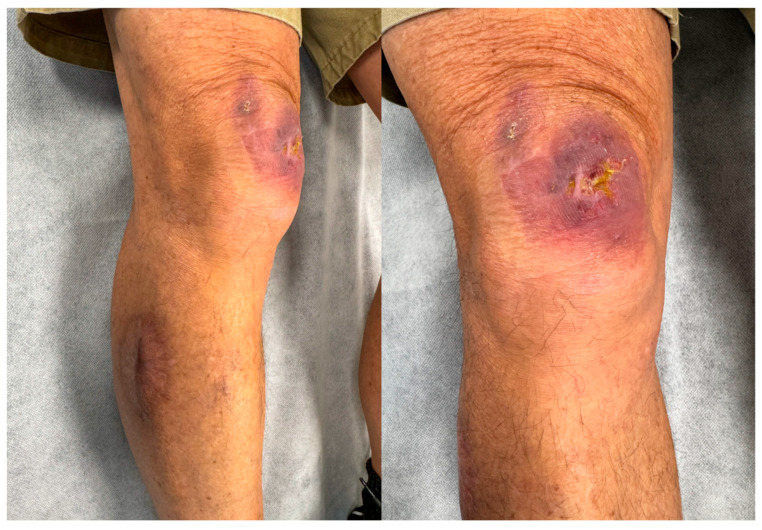
New right knee *M. ulcerans* lesion.

In late March 2024, the patient sustained minor trauma to the left knee, following which he developed a new lesion over the anterior proximal knee. Initially, the lesion was small, painless, and appeared as a non-healing ulcer (Figure 2). Given his prior history of *M. ulcerans* infection and residence in an endemic area, a wound swab was promptly sent for PCR analysis, confirming the presence of *M. ulcerans*. The patient was urgently referred to a plastic surgeon for further evaluation and management.

**Figure 3:**
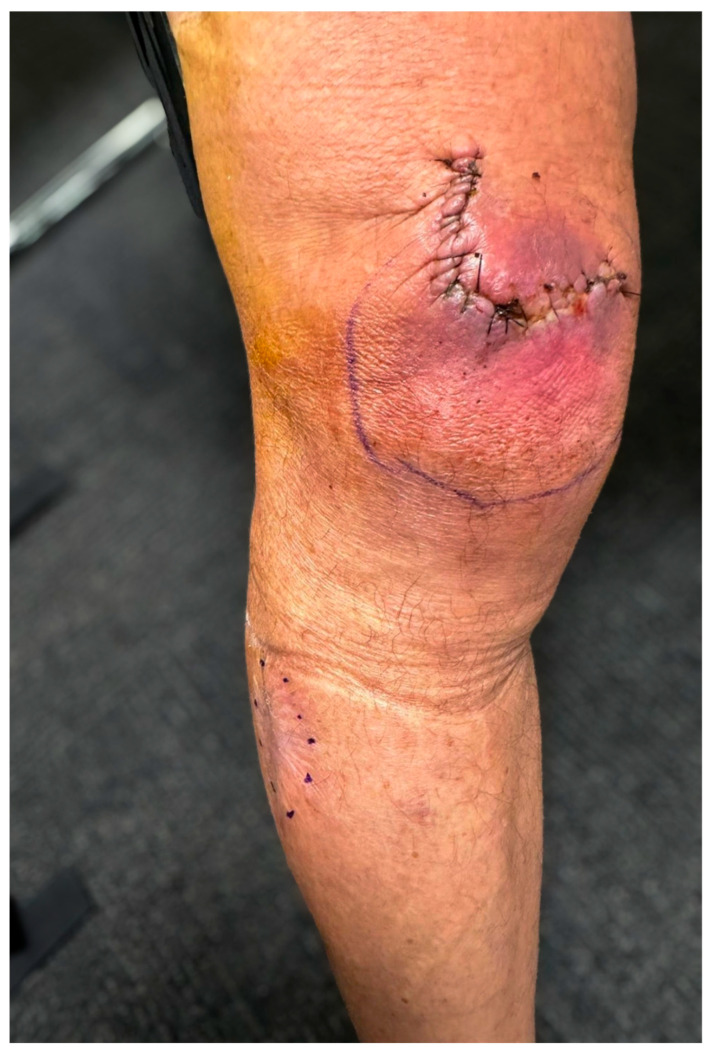
Postoperative clinical photograph of anterior knee *M. ulcerans* ulcer.

**Figure 4:**
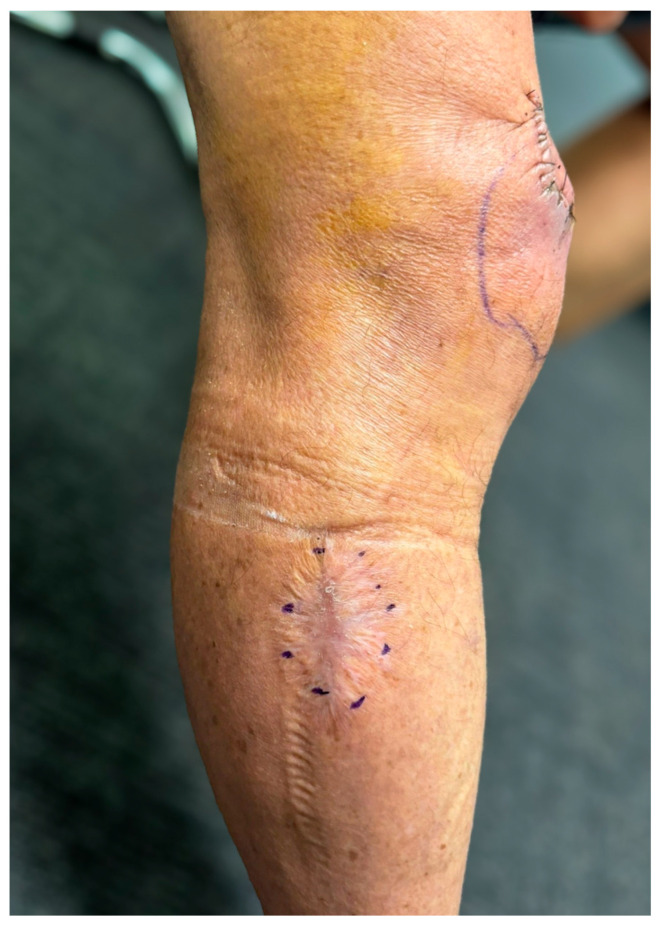
Clinical photograph of close anatomical proximity of right lateral *M. ulcerans* and right anterior knee ulcers.

Biochemical tests, including inflammatory markers, were within normal limits. In July 2024, the patient underwent repeat excisional debridement of the recurrent ulcer at the anterior proximal knee (Figure 3). Intraoperative assessment suggested that while the debridement aimed to achieve clear margins, complete eradication of infected tissue might not have been fully accomplished. Figure 4 highlights the close anatomical relationship between the initial *Mycobacterium ulcerans* lesion and the site of the postoperative debridement. However, intraoperative findings suggested that complete removal of infected tissue might not have been achieved. Consequently, a postoperative course of rifampicin (300 mg twice daily) and clarithromycin (250 mg twice daily) was initiated for 56 days. The antibiotic dose of clarithromycin was adjusted due to his estimated glomerular filtration rate (eGFR) of 41, and his cardiologist approved the regimen, as he was no longer on flecainide. The patient tolerated the antibiotic therapy well. His wound progressed favorably, achieving complete healing with no signs of recurrence. He remains under regular outpatient follow-up with an infectious disease specialist and plastic surgeon.

In our patient, the new lesion appeared seven months after the primary infection. The relatively prolonged healing duration of four months was attributed to the patient’s age, mild chronic kidney disease, and the decision not to use adjunctive antibiotic therapy at the time. The excisional wound was initially managed with moist wound healing techniques using hydrofiber dressings containing ionic silver (e.g., Aquacel Ag), which were changed every 2 to 3 days. Wound progress was monitored weekly by the plastic surgery outpatient team, and secondary intention healing was observed. Delayed epithelialization and low-grade inflammation likely contributed to the extended healing time on the same limb but anatomically separate. While this may suggest a skip lesion via lymphatic or hematogenous spread, the possibility of a new inoculation event must also be considered. The absence of systemic symptoms, the anatomical proximity of the lesions, and the short interval between them supported our clinical suspicion of a skip lesion, yet definitive differentiation was not possible. We acknowledge that without molecular confirmation, such as whole-genome sequencing (WGS), we cannot conclusively determine whether the second lesion was clonally related to the first. WGS enables detailed phylogenetic analysis and has been employed in outbreak investigations and recurrence studies but was not available in our clinical setting. This limitation highlights the need for standardized diagnostic criteria and increased accessibility of molecular tools in endemic areas.

In this case, the unusual presentation of skip lesions prompted further investigation into the unique characteristics of *M. ulcerans* infections, particularly their dissemination patterns. This case underscores the diagnostic and therapeutic challenges associated with Buruli ulcers, emphasizing the need for early recognition, a multidisciplinary approach, and a tailored treatment strategy that balances surgical intervention with appropriate antibiotic therapy.

## 3. Results

The systematic literature review identified 12 peer-reviewed studies that met the inclusion criteria, specifically those that documented skip lesions in *Mycobacterium ulcerans* infections or provided sufficient clinical detail to identify such cases. These studies reported a cumulative total of 1828 individual patients with confirmed *M. ulcerans* infections. Of these, 1179 cases (64.5%) included either direct mention of non-contiguous, anatomically separate lesions, or clinical descriptions consistent with skip lesions. Some studies focused solely on skip lesions, while others included both contiguous and non-contiguous presentations. This proportion should not be interpreted as the global prevalence of skip lesions but rather reflects a selection bias inherent to the review’s inclusion criteria, which specifically targeted atypical or multifocal cases. The geographic distribution of cases included endemic regions in both tropical and temperate climates, such as Congo (Central Africa), Benin (West Africa), Ghana (West Africa), and southeastern Australia, particularly the Bellarine and Mornington Peninsulas (Table 1).

Given the focus of the literature search, it was anticipated that most studies would report on the standard dual antibiotic regimen of rifampicin and clarithromycin, the most commonly used pharmacological therapy. In many cases, this medical management was supplemented by surgical debridement, particularly in patients presenting with extensive necrosis or delayed diagnoses. Common comorbidities noted across the studies included diabetes mellitus, hypertension, hypercholesterolemia, and immunosuppression, although these were inconsistently reported. Where documented, successful outcomes were generally associated with early diagnosis and combined medical and surgical therapy, especially in cases involving multiple or skip lesions.

## 4. Discussion

This case underscores the challenge of distinguishing between recurrence, dissemination, and reinfection in patients with atypical *Mycobacterium ulcerans* presentations. The development of a second lesion at a non-contiguous site raises diagnostic uncertainty, particularly in endemic areas where environmental re-exposure is likely. Infections typically manifest as ulcers or nodular lesions, with the WHO classifying lesions into three categories based on size, location, and number [23]. In our patient, the new lesion appeared seven months after the primary infection, on the same limb but anatomically separate. While this may suggest a skip lesion via lymphatic or hematogenous spread, the possibility of a new inoculation event must also be considered. The absence of systemic symptoms, the anatomical proximity of the lesions, and the short interval between them supported our clinical suspicion of a skip lesion, yet definitive differentiation was impossible. We acknowledge that without molecular confirmation, such as WGS, we cannot determine whether the second lesion was clonally related to the first. WGS enables detailed phylogenetic analysis and has been employed in outbreak investigations and recurrence studies but was not available in our clinical setting. This limitation highlights the need for standardized diagnostic criteria and increased accessibility of molecular tools in endemic areas.

Severe cases are more frequently observed in immunocompromised individuals or those at the extremes of age [7]. In Australia, *M. ulcerans* infections predominantly present as single lesions, though multiple lesions are seen in approximately 5% of patients [10]. This case, featuring a skip lesion pattern, underscores the diagnostic and therapeutic challenges associated with less common presentations, such as multiple, non-contiguous lesions. Notably, Australian strains of *M. ulcerans* exhibit genomic differences from those found in Africa, producing mycolactone C rather than mycolactone AB, which may account for some regional variations in pathogenic characteristics [24]. Skip lesions in *M. ulcerans* infections are infrequently documented in the literature and refer to non-contiguous ulcerative sites along a limb or body area. These lesions are often linked to delayed diagnosis or inadequate treatment, with the leading hypothesis suggesting subclinical bacterial spread that later manifests at distant sites. The pathophysiology underlying skip lesions remains unclear, though proposed mechanisms include hematogenous or lymphatic dissemination [14]. Given that *M. ulcerans* primarily targets subcutaneous tissue, spread via lymphatic or hematogenous routes is a plausible explanation for discrete infections along the same limb. Supporting this theory, recent research using a ringtail possum model of Buruli ulcer demonstrated both lymphatic and hematogenous dissemination of *M. ulcerans* [25,26]. This experimental evidence reinforces the hypothesis that systemic or localized spread contributes to the development of skip lesions.

Additionally, multiple non-contiguous lesions may reflect a more complex infection pathway, potentially involving multiple inoculation sites or increased host susceptibility [13]. Recent studies suggest skip lesions may be more common than previously recognized, especially in patients with delayed diagnosis or incomplete treatment [18,20]. Given the potential for extensive tissue damage and the need for aggressive surgical intervention if mismanaged, the early identification and treatment of skip lesions require a high index of suspicion. Prompt recognition and appropriate therapeutic strategies are crucial to mitigating complications and improving patient outcomes.

The WHO-endorsed standard antimicrobial regimen for *M. ulcerans* infection currently comprises an 8-week course of rifampicin and clarithromycin [8]. This dual-antibiotic approach effectively targets slow-growing mycobacteria, aiming to limit disease progression while preserving surrounding tissue. Recent trials have also suggested a shortened 4-week triple-antimicrobial regimen, with the addition of amoxicillin-clavulanate [21], or a 6-week regimen, both showing promising results [19]. This modification may accelerate bactericidal activity, rapidly reducing local mycolactone levels and allowing an adequate host immune response to clear the infection. Surgical debridement is usually necessary in cases with extensive tissue involvement or when antibiotic therapy alone is insufficient [9].

Furthermore, adjunctive hyperbaric oxygen therapy (HBOT) has been described in the literature as a potential treatment for severe cases, with the ability to enhance wound healing and reduce bacterial load [27]. However, its availability and cost remain limiting factors for widespread use in routine practice.

We hypothesize that in our case, the recurrent lesion was likely present at the time of the initial surgery but remained clinically silent. Despite a documented 95% cure rate with clear histological margins [9], the risk of recurrence persists if multiple lesions, including those at a skip site, are present but not clinically evident at the time of surgery. This underscores the limitations of a surgical-only approach without antibiotics in such cases. Combining conservative surgical excision with adjunctive antibiotic therapy, as employed in this case, aligns with evidence suggesting that a combined approach results in a higher cure rate, even in recurrence or skip lesions [28,29].

This case underscores the importance of early recognition and timely intervention in *M. ulcerans* infections, particularly in endemic areas. Clinicians should maintain a high level of suspicion for Buruli ulcers in patients presenting with chronic, non-healing ulcers, especially those residing in or visiting endemic regions. The occurrence of skip lesions, though rare, should prompt a thorough investigation into possible dissemination patterns, and treatment plans should be adapted accordingly to ensure optimal patient outcomes. This case reinforces the importance of integrating conservative surgical excision with antibiotic therapy to optimize treatment success and reduce recurrence rates in many cases.

## 5. Conclusions

This case report highlights the uncommon presentation of skip lesions in a patient with *Mycobacterium ulcerans* infection, contributing to the limited literature on this atypical manifestation of Buruli ulcer. The diagnostic and therapeutic complexities associated with non-contiguous lesions underscore the need for early recognition and a multidisciplinary approach to management. While *M. ulcerans* infections typically follow a localized pattern, the occurrence of skip lesions suggests alternative mechanisms of bacterial dissemination, including lymphatic spread, hematogenous distribution, or multiple inoculation events. Further research is warranted to elucidate these processes and improve our understanding of the pathogenesis of atypical disease presentations.

## Figures and Tables

**Table 1:** Literature review of all studies of documented skip lesions.

Study	Year	Region	Number of Cases	Skip Lesions Present	Comorbidities	Treatment	Outcome	Comments
Phanzu et al. [11]	2011	Congo, Central Africa	238 with 13 facial	Yes (3)	None reported	Rifampicin + streptomycin +/− surgery	Delayed treatment of facial ulcers led to complications	Early diagnosis improves outcomes, particularly in unexpected lesion locations
Ruf et al. [12]	2011	Benin, West Africa	224	Yes (2)	None reported	Rifampicin + Streptomycin + surgical intervention	Secondary satellite lesions developed during 8-week course	Possible immune response-mediated paradoxical reactions
Friedman et al. [13]	2013	Bellarine Peninsula, Victoria, Australia	43	No	Diabetes, malignancy, immunosuppression	Rifampicin + Ciprofloxacin/Clarithromycin + Surgery	Potential efficacy of shortened antibiotic course (4 weeks)	Genomic differences in *M. ulcerans* strains may affect treatment response
O’Brien et al. [14]	2015	Bellarine Peninsula (Barwon Health)	327	Yes (3)	Elderly population	Rifampicin + Ciprofloxacin + Clarithromycin	Older patients more likely to have multiple lesions	Potential role of immune response in causing multiple lesions
O’Brien et al. [15]	2017	Bellarine Peninsula, Australia	70	Yes (3 cases)	Immunosuppression	Rifampicin + Clarithromycin	Full recovery, but delayed healing in patients with skip lesions	Delayed treatment may lead to skip lesions
Loftus et al. [16]	2019	Victoria, Australia	55	Yes (4 cases)	Hypercholesterolemia	Rifampicin + Clarithromycin	Successful healing in most cases, additional surgical debridement needed	Skip lesions are rare but present; early intervention is key
Tai et al. [17]	2019	Ghana, West Africa	320	No	Immunosuppression	Rifampicin + Streptomycin	Significant healing in all cases, no recorded skip lesions	Study focused on single lesions, low skip lesion occurrence
O’Brien et al. [18]	2020	Victoria, Australia	85	Yes (5 cases)	Diabetes, hypertension, immunosuppression	Rifampicin + Clarithromycin + Surgery	Good recovery, delayed healing in 2 cases with skip lesions	Skip lesions associated with delayed treatment
O’Brien et al. [19]	2020	Australia (coastal areas)	100	No	Hypertension, diabetes	Rifampicin + Moxifloxacin + Surgery	Successful recovery, no cases of skip lesions recorded	Environmental factors may play a role in increasing incidence
Yerramilli et al. [20]	2021	Victoria, Australia	120	Yes (3 cases)	Hypertension, hypercholesterolemia	Rifampicin + Clarithromycin	Full recovery in most cases, surgical debridement required	Mornington Peninsula cases attributed to environmental factors
Johnson et al. [21]	2022	Benin, West Africa (BLMS4BU trial)	186	No	HIV/TB	Rifampicin + Clarithromycin for 8 weeks OR Amoxi/clav for 4 weeks	Ongoing trial, potential for shortened 4-week triple therapy course	Study suggests bactericidal activity prevents spread
Vandelannoote et al. [22]	2023	Mornington Peninsula, Australia	60	Yes (4 cases)	Hypertension, GORD	Rifampicin + Clarithromycin	Full resolution with combined medical and surgical management	Possum feces identified as a potential environmental source

## Data Availability

The original contributions presented in this study are included in the article. Further inquiries can be directed to the corresponding author.

## Abbreviations

The following abbreviations are used in this manuscript:

BLMS4BU	Buruli Lesion Management Study for Buruli Ulcer;
BU	Buruli ulcer;
eGFR	Estimated Glomerular Filtration Rate;
GORD	Gastroesophageal Reflux Disease;
HBOT	Hyperbaric Oxygen Therapy;
HIV	Human Immunodeficiency Virus;
*M. ulcerans*	*Mycobacterium ulcerans*;
PCR	Polymerase Chain Reaction;
TB	Tuberculosis;
WHO	World Health Organization.
